# Films Based on Mater-Bi^®^ Compatibilized with Pine Resin Derivatives: Optical, Barrier, and Disintegration Properties

**DOI:** 10.3390/polym13091506

**Published:** 2021-05-07

**Authors:** Miguel Aldas, Cristina Pavon, José Miguel Ferri, Marina Patricia Arrieta, Juan López-Martínez

**Affiliations:** 1 Instituto de Tecnología de Materiales (ITM), Universitat Politècnica de València (UPV), 03801 Alcoy, Spain; crisppavonv@gmail.com (C.P.); joferaz@upvnet.upv.es (J.M.F.); 2 Departamento de Ciencia de Alimentos y Biotecnología, Facultad de Ingeniería Química y Agroindustria, Escuela Politécnica Nacional, Quito 170517, Ecuador; 3 Departamento de Ingeniería Química y del Medio Ambiente, Escuela Técnica Superior de Ingenieros Industriales, Universidad Politécnica de Madrid (ETSII-UPM), Calle José Gutiérrez Abascal 2, 28006 Madrid, Spain; m.arrieta@upm.es; 4 Grupo de Investigación: Polímeros, Caracterización y Aplicaciones (POLCA), 28006 Madrid, Spain

**Keywords:** thermoplastic starch, pine resin, colophony, gum rosin, pentaerythritol esters of gum rosin, compostable, biodegradable

## Abstract

Mater-Bi^®^ NF866 (MB) was blended with gum rosin and two pentaerythritol esters of gum rosin (labeled as LF and UT), as additives, to produce biobased and compostable films for food packaging or agricultural mulch films. The films were prepared by blending MB with 5, 10, and 15 wt.% of each additive. The obtained films were characterized by optical, colorimetric, wettability, and oxygen barrier properties. Moreover, the additives and the MB-based films were disintegrated under composting conditions and the effect of each additive on the biodegradation rate was studied. All films were homogeneous and optically transparent. The color of the films tended to yellow tones due to the addition of pine resin derivatives. All the formulated films presented a complete UV-transmittance blocking effect in the UVA and UVB region, and those with 5 wt.% of pine resin derivatives increased the MB hydrophobicity. Low amounts of resins tend to maintain the oxygen transmission rate (OTR) values of the neat MB, due to its good solubilizing and compatibilizing effects. The disintegration under composting conditions test revealed that gum rosin completely disintegrates in about 90 days, while UT degrades 80% and LF degrades 5%, over 180 days of incubation. As expected, the same tendency was obtained for the disintegration of the studied films, although Mater-Bi^®^ reach 28% of disintegrability over the 180 days of the composting test.

## 1. Introduction

Biodegradable plastics films have risen as a solution for the use of non-renewable resources and the solid waste disposal management of conventional non-biodegradable polymers when recycling is impractical or not economical [1,2,3]. Biodegradable plastics are typically derived from renewable materials such as starch or cellulose and can be disposed by composting or anaerobic digestion to reduce landfilling [4,5].

Starch-based plastics have gained great attention due to their biodegradability, renewability, and low cost [6,7]. Starch is a carbohydrate composed of linear polysaccharide molecules (amylose) and branched polysaccharide molecules (amylopectin) [7,8]. Starch can be plasticized during extrusion, where the combination of shear, temperature, and plasticizers allows the disruption of the native crystalline granular structure leading to an amorphous plasticized starch so-called thermoplastic starch (TPS) [3,9]. Thermoplastic starch offers the advantage that it can be processed by extrusion, injection molding, or thermoforming as conventional synthetic plastics [3,7]. However, TPS has some disadvantages such as high hydrophilicity, poor mechanical performance, and an increase in brittleness with time due to amylose recrystallization (retrogradation) [3,8,10]. These limitations restrict the use of TPS in some applications such as food packaging or disposable films [11]. Therefore, TPS is generally commercialized in blends with hydrophobic polymers or with less hydrophilic biodegradable polymers, to attain a biodegradable material with enhanced properties with affordable costs for consumers [12,13,14]. In this context, thermoplastic starch blended with synthetic polymers (such as aliphatic polyesters) is nowadays commercialized by different companies; for instance Novamont (Novara, Italy) or Biotec (Emmerich am Rhein, Germany) [15]. Novamont Mater-Bi^®^ is a family of biodegradable and/or compostable polymeric formulations containing thermoplastic starches, which have good thermal stability and processability, and present high stretchability and toughness [12,16,17].

The Mater-Bi^®^ family is composed of different grades of thermoplastic starches with different properties whose composition varies mainly in the type and amount of synthetic polymer used [18,19]. For instance, Mater-Bi^®^ A is based on copolymers of polyvinyl alcohol (PVA) and it is not compostable. Mater-Bi^®^ V is formed of higher contents of TPS (greater than 85%) and has high water solubility. Mater-Bi^®^ Y is blended with cellulose acetate and its properties resemble those of polystyrene. In Mater-Bi^®^ Z poly(ε-caprolactone) (PCL) is employed as synthetic polymer and Mater-Bi^®^ N uses poly(butylene adipate-co-terephthalate) (PBAT) as the synthetic polymer [13,18,19]. In this work, Mater-Bi^®^ NF866 based on TPS, PBAT, and PCL blend was chosen as its properties allow it to obtain thin films to be used as an alternative to polyethylene in the agricultural and packaging industries [20].

The interest in the biodegradable materials field is also increasing in the terms of the additives used in plastics production processes, to assure the eco-friendly nature of the final product [21,22]. Gum rosin (GR) and pine resin derivatives are attractive and low-cost renewable alternatives that have great potential in the development of blends with biopolymers [19,23,24]. Gum rosin is the non-volatile fraction of pine resin; it is a rigid and brittle solid composed primarily of abietic- and pimaric-type rosin acids [23,25,26]. The structure of rosin acids has conjugated double bonds and a carboxylic group, which allow its modification into salts, esters, and hydrogenated rosin [25,27,28]. In this sense, pentaerythritol esters of gum rosin are low/medium molecular weight gum rosin ester derivatives (between 800–3000 Da) mainly enriched with tri- and tetra-ester functional groups, which possess lower acid number (below 30 mgKOH/g) than gum rosin (around 160 mgKOH/g) [29]. Pentaerythritol rosin esters have shown several advantages for the plastic processing industry such as the high softening point (between 80 and 120 °C) [29,30] with improved heat stability as well as with low odor and volatility [27]. The ester form of rosin enables to provide high hydrophobic performance to biopolymeric polymeric matrices due to its reduced acid number [19]. These esters are widely used in paints, varnishes, adhesives, packaging, and drug microcapsules [27,31]. Moreover, pentaerythritol gum rosin esters are ecological and approved by the FDA [31], and thus are gaining interest in the sustainable food packaging sector [19,30,32,33,34].

**Figure 1:**
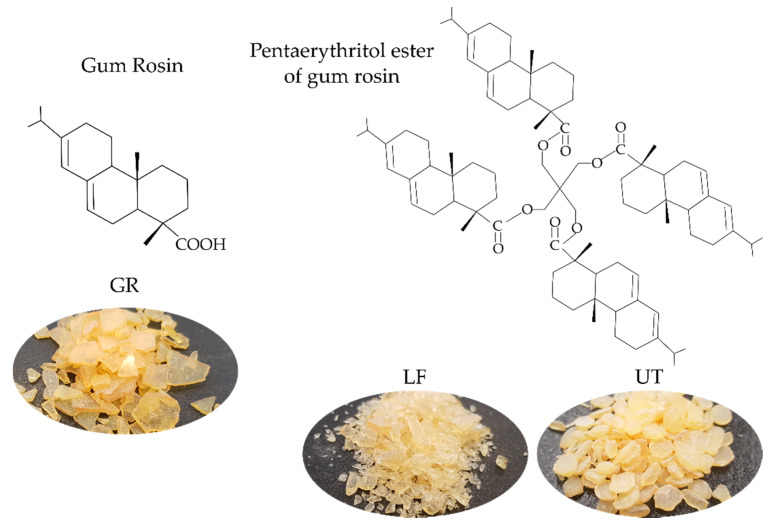
Chemical structure of the pine resin derivatives employed in the study.

In a previous work, the effect of different chemical modifications of gum rosin (disproportionated gum rosin, maleic anhydride-modified gum rosin, glycerol ester of gum rosin, and pentaerythritol ester of gum rosin) on TPS properties was studied. Among all these modified gum rosins, it was observed that the pentaerythritol ester of gum rosin (Figure 1) produced a significant stiffening effect and good thermal stability, due to the higher amounts of carbonyl groups in its chemical structure able to interact with the TPS hydroxyl groups [32]. Moreover, the effect of gum rosin (GR), Lurefor (LF), and Unik Tack (UT) (chemical structure in Figure 1) on the processability, thermal, and mechanical properties of injection molded Mater-Bi^®^ NF866 were studied and it was determined that the thermal and mechanical properties of Mater-Bi can be adjusted by varying the type and amount of gum rosin additive thanks to the plasticizing, solubilizing, and/or compatibilizing effect of the resins [30]. These findings were further corroborated by focusing on the microstructure of the blends which provided a better understanding of the Mater-Bi^®^ NF866 structure and components (TPS, PBAT, and PCL) and the effect of the gum rosin additives. It was also revealed that the formulations with gum rosin and pine resin derivatives had improved miscibility and solubility of the components of the matrix. This improvement is directly linked to the used additives [19].

The present work aims to expand the study of Mater-Bi^®^-based materials compatibilized with pine resin derivatives and explore their properties to assess the potential application in the field of compostable films for food packaging or agricultural mulch films. For this purpose, Mater-Bi^®^ NF866 (MB) was melt-blended with GR, and two pentaerythritol esters of gum rosin: LF (softening point of 125 °C, acid number 11.9, Gardner color: 7), and UT (softening point of 90 °C, acid number 15, Gardner color: 4) simulating the industrial processing conditions and further processed into films by means of the compression molding process. The MB-resin formulations were prepared by melt blending MB with 5, 10, and 15 wt.% of each additive (pine resin derivatives), separately. To propose these formulations for food-related applications, the obtained films were characterized in terms of visual appearance, optical, colorimetric properties, surface wettability as well as UV and oxygen barrier performance. Finally, the disintegration of the blends under composting conditions was assessed at a laboratory scale to get information about the effect of pine resin derivatives on the disintegration rate of MB polymeric matrix.

## 2. Materials and Methods

### 2.1. Materials

Mater-Bi^®^ NF 866 (labeled as MB) was supplied by Novamont SPA (Novara, Italy). MB is a commercial material based on starch and aliphatic-aromatic polyesters (PBAT and PCL). The additives mixed with MB were gum rosin or colophony (labeled as GR, softening point of 76 °C and acid number 167), supplied by Sigma-Aldrich (Mostoles, Spain), and two pentaerythritol esters of gum rosin: Unik Tack P100 resin (softening point of 90 °C, acid number 15, Gardner color: 4), labeled as UT, supplied by United Resins (Figueira da Foz, Portugal) and Lurefor 125 resin (softening point of 125 °C, acid number 11.9, Gardner color: 7), labeled as LF, supplied by LureSA (Segovia, Spain). LF and UT differ each other in the acid number, which denotes that the number of functionalized groups in the modified structure is greater in LF than in UT [23,35,36]. Besides, they have different color due to the different levels of chemical modifications [37].

### 2.2. Film Preparation

All materials were conditioned at 25 ± 1 °C and 50 ± 5% RH for 24 h before being processed. Then, MB was melt-blended with pine resin derivatives in 5, 10, and 15 wt.%, in a twin-screw extruder (Dupra S.L, Castalla, Spain), using a temperature profile of 160 °C, 150 °C, 140 °C, 100 °C (from die to hopper) at 50 rpm. The blends were then milled into pellets to be further molded into films at 170 °C in a hot press (Mini C 3850, Caver Inc., Atlanta, TX, USA). The blends were kept between the plates at atmospheric pressure for 5 min until melted and then submitted to the following pressure cycle, 3 MPa for 1 min, 5 MPa for 1 min, and 10 MPa for 3 min, to liberate trapped air bubbles. Film samples were then quenched to room temperature at atmospheric pressure. Squared films (14 × 14 cm^2^) of 450 μm of thickness were obtained and they were labeled according to Table 1.

### 2.3. Film Characterization

#### 2.3.1. Structural, Visual Appearance, and Optical Properties

The visual appearance of all obtained films was evaluated by simple inspection. Thus, the films were assessed by placing each film over a printed pattern and taking photographs. The light transmission of MB-based blends was determined by UV-Vis spectroscopic analyses carried out on a Cary 100 UV-Vis spectrophotometer by Agilent technologies (Barcelona, Spain). The test was carried out at a range between 250 to 700 nm to evaluate the optical changes due to the addition of pine resin derivatives in Mater-Bi. The UV-Vis absorption spectra of Mater-Bi and Mater-Bi-pine resin derivatives films were obtained. The spectra of each formulation were normalized to film thickness (400 µm).

Film color properties were assessed by measuring color coordinates in the CIELAB color space L (lightness), a* (red-green), and b* (yellow-blue), using a Konica CM-3600d Colorflex-Diff2 458/08 colorimeter from HunterLab, Hunter Associates Laboratory Inc., (Reston, VA, USA). The instrument was calibrated with a black and white standard tiles. Then, 10 different measurements for each film were obtained at random positions and the average values are reported. The Total Color Differences (ΔE*_ab_), induced by the resins into the MB film was evaluated according to Equation (1), using neat MB film as reference. The yellowness index (YI) was also measured and reported.

(1) $$\Delta E^{*}{}_{ab}=\sqrt{\Delta a^{2}+\Delta b^{2}+\Delta L^{2}},$$

The studied materials, obtained films, and starting materials (resins and MB matrix) were also assessed by attenuated total reflectance Fourier transform infrared spectroscopy (FTIR), using a Perkin Elmer Spectrum BX (FTIR system). The spectra of the films were obtained directly from the sample, while the spectra of resins were recorded in transmission mode, using the KBr discs technique. The results were recorded within the range of 4000–650 cm^−1^, with a resolution of 4 cm^−1^ and 32 scans. Scanning electron microscopy (SEM) was performed using a Phenon SEM equipment of FEI (Eindhoven, The Netherlands) with a voltage of 5 kV and using a working distance of 60.2 μm for 4000× of magnification. The images were obtained from the cryofractured coated surface, coated with a gold-palladium alloy on a Sputter Mod Coater Emitech SC7620, Quorum Technologies (Lewes, East Sussex, UK).

#### 2.3.2. Wettability

Wettability was assessed employing the water contact angle (WCA), measuring the static contact angle, Theta (θ), of a drop created on the surface of the films. The measurements were conducted at room temperature using an EasyDrop-FM140 optical goniometer FM140 from Kruss equipments (Hamburg, Germany). The goniometer was equipped with a camera and the Drop Shape Analysis software. Ten contact angle measurements were taken in random positions, putting 2 μL drops of distilled water onto the surface of films with the aid of a syringe. The average values of the WCA were calculated and reported.

#### 2.3.3. The Oxygen Transition Rate

The oxygen transition rate (OTR) measurements were assessed using an oxygen permeation analyzer from Systech Instruments Model 8500 (Metrotec S.A, San Sebastián, Spain) at a pressure of 2.5 atm. Three samples were assessed at room temperature. Films were clamped in the diffusion chamber and pure oxygen (99.9% purity) was flowed through the upper half of the sample chamber, while nitrogen flowed through the lower half of the chamber. The results were expressed as oxygen transmission rate per film thickness (OTR·*e*). The thickness of films (*e*) was determined at 25 °C using a Kalkum Ezquerra SL Micrometer (La Rioja, Spain) with 0.001 mm accuracy, from 10 readings at random positions. The shape of films for OTR assessment was 14 cm diameter circle films.

#### 2.3.4. Disintegration under Composting Conditions

Disintegration under composting conditions test was performed by following the ISO-20200 standard [38] at a temperature of 58 °C and relative humidity of 55%. Samples sizing 10 × 10 mm^2^ were placed in a carrier mesh and buried 6 cm depth in plastic reactors containing the solid synthetic wet waste manufactured following ISO-20200. Reactors were introduced in an air circulation oven at 58 °C for 90 days for thermophilic degradation. The aerobic conditions were guaranteed by periodical gentle mixing of the solid synthetic wet waste [39]. After 90 days of the test, 25 g of compost was added to the reactors and the oven temperature was change to 25 °C for additional 90 days to assess the mesophyll degradation. In this stage, the solid synthetic waste was not mixed. Before placing samples into the reactor, all films were dried at 40 °C for 48 h. Film samples were extracted at 1, 4, 7, 11, 14, 21, 42, 90, 135, and 180 days. In each extraction, the samples were washed with distilled water and dried again at 40 °C for 48 h. Disintegrability (D, in %) was calculated by normalizing the sample weight at each time (m_r_) to the initial value (m_i_) as shown in Equation (2), while photographs were taken to qualitative follow the disintegration process. The disintegration process of gum rosin (GR) and both pentaerythritol ester of gum rosin (UT and LF) were also evaluated. Thus, films of each pine resin were prepared by melting each additive over its softening point and putting them in molds to further let them cool down to room temperature.

(2) $$D=\frac{m_{i}-m_{r}}{m_{i}}\times 100$$

#### 2.3.5. Statistical Analysis

Significance differences in color, WCA, and OTR measurements were statistically analyzed using one-way analysis of variance with OriginPro 8 software. Significant differences among the measurements were recorded at a 95% confidence level according to Tukey’s test.

## 3. Results and Discussion

### 3.1. Structural and Optical Properties

**Figure 2:**
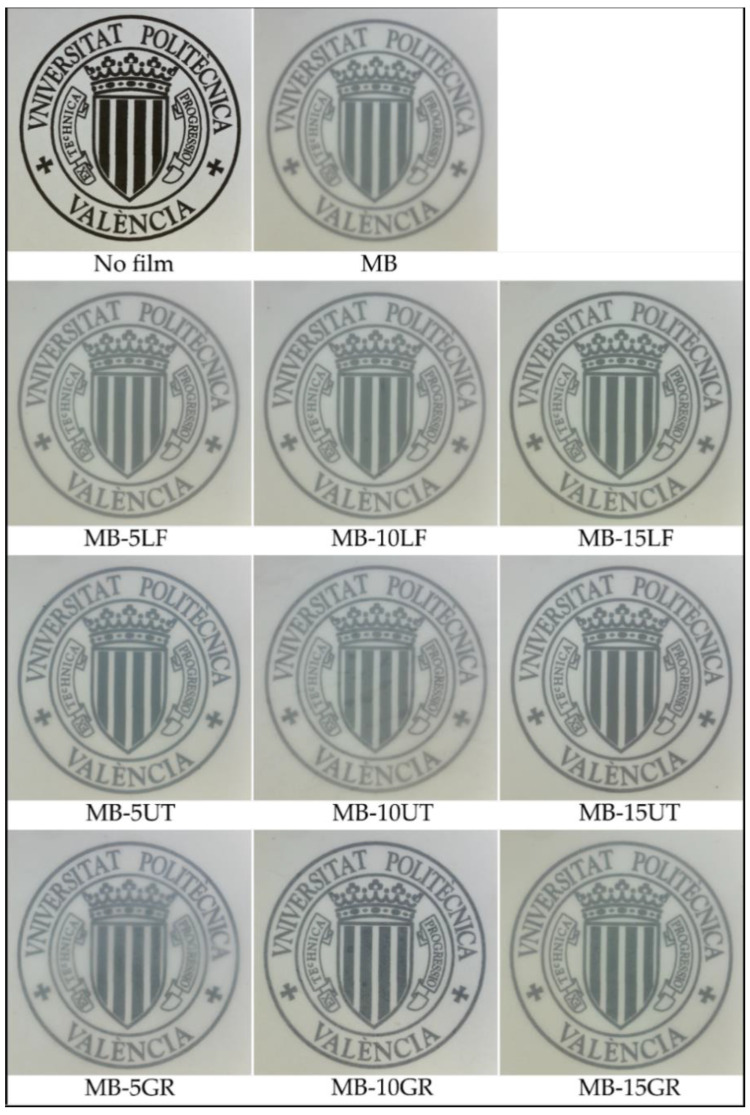
The visual appearance of MB film and its blends with pine resin derivatives.

The processing conditions used here allowed to obtain homogeneous and transparent films with thickenss in the range of 400 ± 10 µm. Figure 2 shows the visual appearance of MB films obtained with different contents of pine resin derivatives (LF, UT, and GR). It is observed that MB and MB-pine resin derivatives films are visually homogeneous regardless of their composition. The homogeneous distribution of pine resins in MB polymeric matrix was corroborated by FESEM and all film formulations resulted mostly homogenous with no phase separation (Figure S1 in the Supplementary Materials), in good agreement with injection molded MB-based materials developed in previous works [19,30]. All the films were clear and translucent, which is a desirable physical property in films intended for food packaging applications, where the consumers want to see the packed product through the packaging [40,41,42]. The cloudy character of the films was attributed to light scattering at the interface of crystalline-amorphous regions of thermoplastic starch [43]. Moreover, it was observed that the color distribution in each film was uniform which suggests that pine resin derivatives are homogeneously distributed in the MB matrix.

Table 2 summarizes the CIELAB color coordinates (La*b*) as well as the yellowness index (YI) and the color differences (ΔE*_ab_) of the pine resin derivatives (GR, UT, and LF) and the formulated blends MB-LF, MB-UT, and MB-GR compared to neat MB film. Two different statistical analyses were done, one among the three pine resin derivatives and the other one among the MB formulates blends. Regarding the pine resin derivatives, it is observed that LF and UT present no significant differences (*p >* 0.05) in L (luminance), while GR has a significantly lower L coordinate. Besides, the a* coordinate (green to red) for LF and UT suggests that neither green nor red is predominant in these samples. On the contrary, GR does present a significant reddish hue. Moreover, the b* coordinate (blue to yellow) indicate that all pine resin derivatives have a yellowish coloration and present significant differences among them (*p <* 0.05). The yellowness index (YI) is in good agreement with the b* coordinate results in these samples. Concerning the MB formulated blends, it is observed that L presents no significant differences (*p >* 0.05) with the addition of 5 wt.% of either LF or UT. However, higher contents (10 or 15 wt.%) of LF or UT significantly (*p <* 0.05) reduces L. The addition of GR has a more intense reduction of L in all the tested compositions. About the a* coordinate, no statistical differences (*p >* 0.05) were detected among the studied materials. In contrast, the b* coordinate of MB significantly increased (*p <* 0.05) due to the addition of pine resin derivatives. This increase is indicative of deviation towards a yellow coloration and is directly proportional to the pine resin content. Moreover, the yellow coloration of MB blends is directly influenced by the b* coordinate of the respective pine resin derivative used, as the yellowish hue value of the pine resin derivatives is high in comparison to that of MB. MB-GR blend presents the higher b* because GR inherent yellow coloration is stronger than that of its esterified derivatives, as seen in Table 2. YI shows a similar tendency to b*. MB-5LF and MB-5UT have values of ΔE*_ab_ smaller than 2, which suggests that the difference is not appreciable for the human eye in these blends [44]. On the other hand, all MB-GR blends have major color differences concerning neat MB, thus it can be troubling to use these blends in food packaging applications. On the other hand, MB-15LF, MB-15UT, MB-5GR, MB-10GR, and MB-15GR present ΔE*_ab_ higher than 5, which indicates that the color difference can be noticed by an inexperienced observer [45].

**Figure 3:**
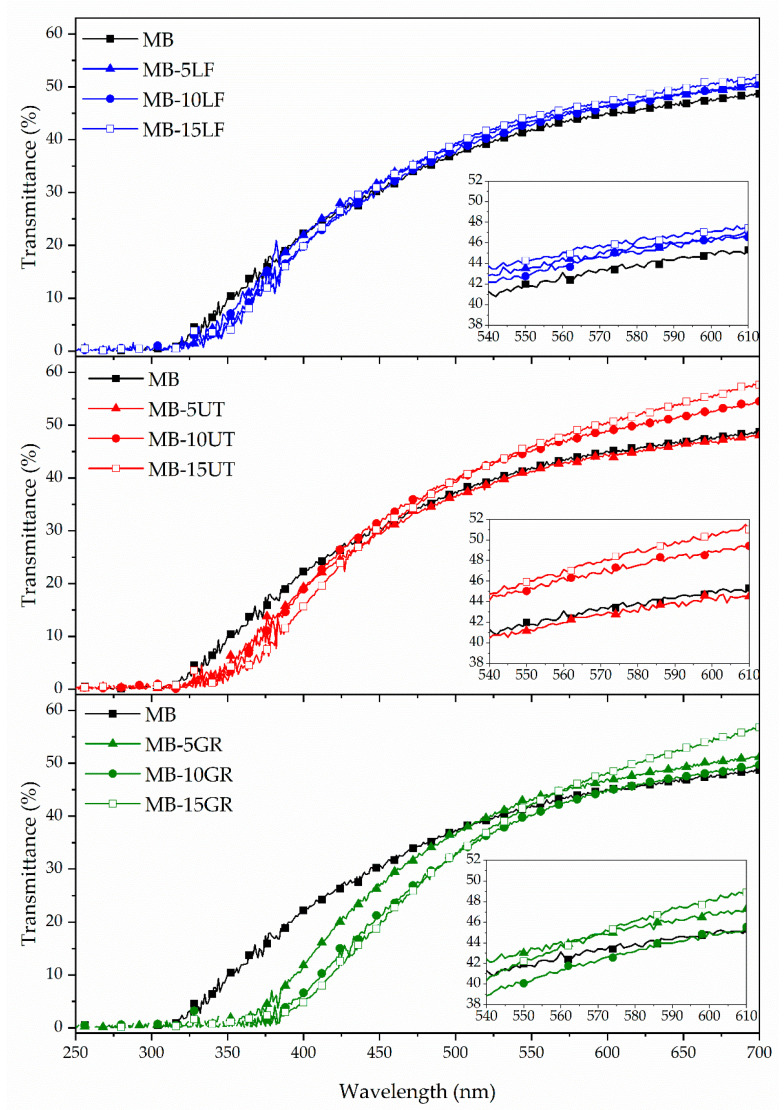
Transmittance spectra (250–700 nm) of films based on Mater-Bi (MB) and MB with 5, 10, and 15 wt.% of pine resin derivatives (LF, UT, and GR, respectively). The thickness of the films was normalized to 400 µm.

Transparency is a very important concern for consumer acceptance in materials intended for food packaging applications. Figure 3 presents the UV spectra of MB and MB- pine resin derivatives blend films. It is seen that all the films present a complete radiation-blocking between 250 to 320 nm (UVB region). It is observed that in the UVA (320 to 380 nm) region, all pine resin derivatives produce a protective effect against UV radiation linked to the presence of aromatic compounds [46] confirming the UV absorption potential of pine resins [47], GR being the additive that offers the greatest UV protection and LF the one with the lowest UV protection. Additionally, the complete UV radiation-blocking effect in this zone is directly dependent on the pine resin content. These results are in good agreement with Narayanan et al. who studied polylactic acid (PLA) blended with an increasing amount of rosin and observed that an increasing rosin content tends to greatly decrease transmittance in all the UV regions [47]. MB-GR films have complete radiation blocking in the UVA region when GR is added in 15 wt.%. MB-10GR has a complete radiation-blocking until 370 nm, and MB-5GR presents a complete radiation-blocking until 355 nm. In the visible region of the spectra (380 to 700 nm), it is observed that until 550 nm, pine resin derivatives act as light-absorbing agents and prevent the transmission of light through the films. Again, GR provides the highest UV protection, while LF shows almost no changes compared to the spectrum of MB. Finally, from 550 to 700 nm no major changes are observed in the spectra of the films with respect to the MB spectrum, confirming once again the homogeneity of the blend formulations due to the homogenous dispersion of the resin additives [48]. The UT resin showed a slight increase in transparency when it was added in high amounts of 10 and 15 wt.%. This slight improvement can be related to the good interaction of UT with the MB polymeric matrix due to the formation of hydrogen bonding interactions between the carbonyl groups of resin and the –OH groups of starch in MB. In this formulation, the carbonyl group peak was shifted from 1730 cm^−1^ in UT neat resin to 1718 cm^−1^ in MB-UT. Meanwhile, in MB-LF-based materials, there are two peaks in this region, and in MB-GR films there was a displacement from 1696 cm^−1^ in GR neat resin to 1682 cm^−1^ in MB-GR-based formulations in a broader peak (Figure S2).

### 3.2. Wettability

**Figure 4:**
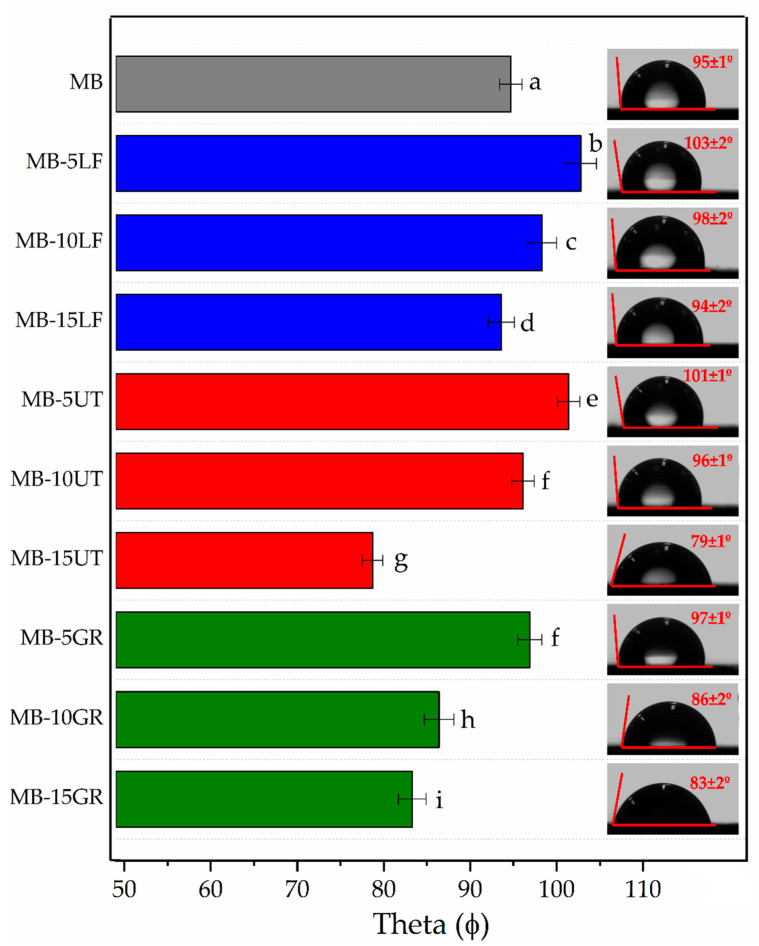
Water contact angle (WCA) measurements of MB and MB- pine resin derivatives films. ^a–i^ Different letters show statistically significant differences between formulations (*p <* 0.05).

Water contact angle (WCA) comparison between MB-based films is presented in Figure 4. In general, high values of WCA were obtained (>65°) for all the films, indicating that MB displays a hydrophobic character. According to the literature, WCA higher than 65° are indicative of hydrophobic surfaces, while WCA lower than 65° are indicative of hydrophilic surfaces [49,50]. All the films exhibit significant differences (*p <* 0.05) in WCA with respect to neat MB. It is seen that pine resin derivatives (GR, LF, and UT) tend to increase MB surface hydrophobicity when added in 5 wt.%.

GR significantly increases MB hydrophobicity in a content of 5 wt.% due to the hydrophobicity of resin and the increased roughness of the surface (see Figure S3) [33]. However, when the content is raised to 10 or 15 wt.%, MB hydrophobicity is significatively reduced (*p <* 0.05). Increasing the GR content in the formulation leads to a higher amount of carboxylic end groups in the formulation able to interact with water at the surface. It should be considered that the surface contact angle measurements are not only dependent on the surface chemical properties, but also on the topographical properties of the surface and the higher amount of pine resin could lead to a flatter surface (not shown). In fact, in previous works [19,30], it was demonstrated that GR acts as a solubilizer in an MB polymeric matrix. Therefore, the interaction between GR and the polymer chains decreases the polymer–polymer interactions among the components of the polymeric matrix, particularly when it was added in high amounts (i.e., 10 and 15 wt.%). LF and UT in 5 and 10 wt.% significantly increase (*p <* 0.05) MB hydrophobicity. The esters groups present in LF and UT (Figure 1) are more hydrophobic than the corresponding carboxylic acids present in GR [34]. Moreover, the hydrophenanthrene rosin moieties of the esters may be located on the surface of the films with the ester groups embedded inside the films [51,52] which increases hydrophobicity. On the other hand, LF and UT in 15 wt.% produce a decrease in MB hydrophobicity. This behavior is linked to the increment of the solubility of resin into the polymeric matrix which leads to a homogeneous and a flater surface, as discuss above with GR results, as UT at 15 wt.% favors the solubility on MB components [19]. MB‑15LF increases the miscibility of MB components [19], which could increase the interactions between the hydrophobic groups showing less reduction on MB surface hydrophobicity.

### 3.3. OTR Evaluation

**Figure 5:**
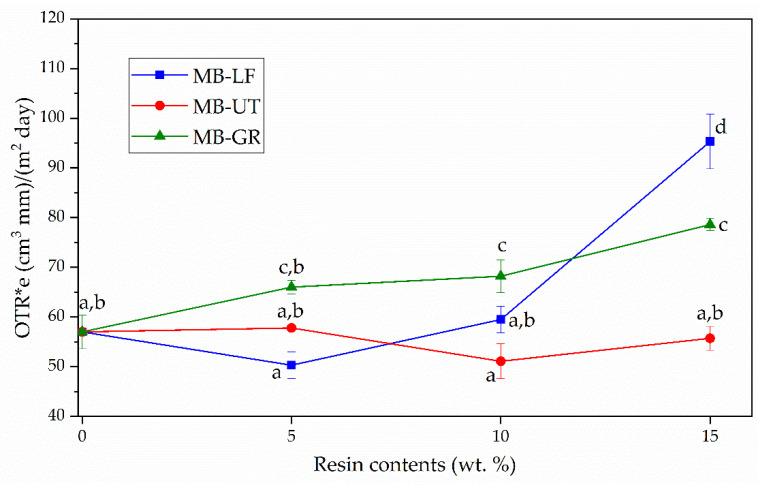
OTR of MB- pine resin derivatives films as a function of pine resin content. ^a–d^ Different letters show statistically significant differences between formulations (*p <* 0.05).

The OTR measurement values obtained for the studied formulations are shown in Figure 5. In general, it is observed that there are large differences between the formulations additivated with GR (unmodified gum rosin) and those with UT and LF (pentaerythritol esters of gum rosin). In particular, the MB-GR formulations are slightly more permeable to O_2_ regarding the MB matrix, varying from 57.0 ± 3.4 (cm^3^·mm)/(m^2^·day) to values between 66.0 ± 1.4 (cm^3^·mm)/(m^2^·day) for MB-5GR and 79.3 ± 1.5 (cm^3^·mm)/(m^2^·day) for MB-15GR. The plasticizing effect exerted by the GR resin on MB causes the free volume between polymer chains to increase and allows greater oxygen transfer [19]. The formulations MB-10UT and MB-5LF show lower OTR values (51.1 ± 3.5 and 50.3 ± 2.7 (cm^3^·mm)/(m^2^·day), respectively) than those obtained for non-additivated MB, indicating that the UT and LF resins act as compatibilizing agents, thus maximizing the interaction between the MB components and improving the polymer barrier performance. In previous works, low compatibility between MB components (TPS, PBAT, and PCL) was evidenced [19,30], which generates discontinuities through the material and the O_2_ transmission is facilitated, increasing the permeability of the material. Therefore, the homogeneous distribution of UT and LF resins into the polymeric matrix and the positive chemical interactions established among the components (i.e., hydrogen bonds), reduce this type of discontinuities and consequently, the oxygen transmission through the film. UT resin was able to maintain the OTR. *e* values despite the amount of resin added, while LF was able to maintain this value in contents lower than 10 wt.%. UT resin possesses a lower softening point (90 °C) than LF (125 °C) and thus allowed a more homogeneous distribution of the resin into the polymeric matrix during processing, which finally leads to better chemical interactions.

The OTR.*e* values obtained here for MB-resin blends are higher than high oxygen barrier polymers such as polyethylene terephthalate (PET) (OTR.*e* < 3 (cm^3^·mm)/(m^2^·day), but lower than that of commercial low-density polyethylene (LDPE) (OTR.*e*: 160–240 (cm^3^·mm)/(m^2^·day), which is a widely used polymer in food packaging and agricultural applications [44].

### 3.4. Disintegration under Composting Conditions Assessment

**Figure 6:**
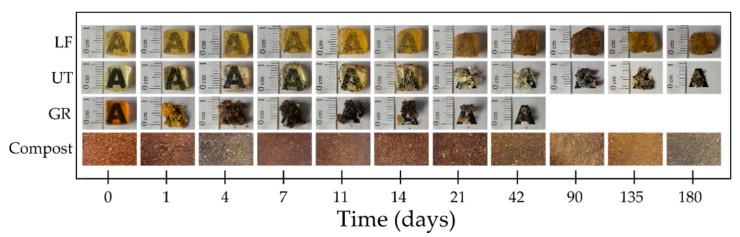
The visual appearance of pine resin derivatives samples recovered at different days of incubation under composting conditions and the evolution of compost medium during the test.

Comparative images of the disintegration under composting conditions in different incubation days of GR, LF, and UT are shown in Figure 6. It is observed that GR is fragmented from day 7 and is disintegrated before 90 days, that is during the thermophilic disintegration period [38]. On the other hand, UT shows fragmentation from day 21 and does not exceed 90% of disintegration until the 180 days of the test. Finally, LF does not present fragmentation of its structure during the 180 days of incubation. The obtained results suggest that both pentaerythritol esters of gum rosin (LF and UT) are more stable and less prone to hydrolytic degradation than unmodified gum rosin, due to the chemical modifications in their structure that lead to higher softening points (LF softening point = 125 °C and UT softening point = 90 °C) than that of GR (GR softening point = 76 °C) [30,32]. Moreover, LF takes a longer time to disintegrate than UT and GR, which is attributed to the availability of hydroxyl groups, as LF presents a lower acid number (LF acid number = 11.9) than UT (UT acid number = 15). Consequently, LF presents fewer hydroxyl groups available for the hydrophilic interaction with water than UT, which delays its capacity to start the hydrolytic mechanism. Moreover, the fact that LF also possesses the highest softening point leads to the stiffest material which is more resistant to the overall disintegration processes due to the reduced molecular mobility. For this reason, LF is the resin that takes the longest time to disintegrate.

**Figure 7:**
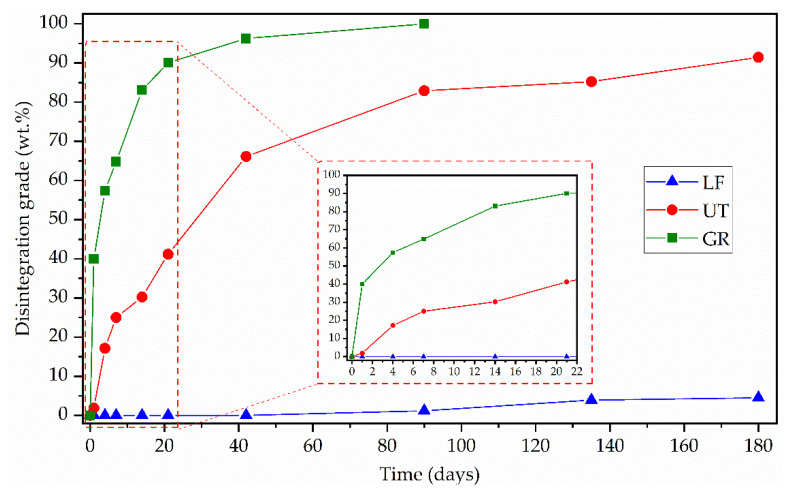
Disintegration degree of the samples of pine resin derivatives at different days of incubation under composting conditions.

These findings were corroborated by determining the quantitative disintegration degree concerning the incubation time for the studied pine resin derivatives (Figure 7). In this figure, the difference in the disintegration rate of each resin can be better appreciated. GR degrades 95% at day 42 and completely disintegrates before 90 days of incubation under simulated composting conditions. UT degrades 80% at the end of the test (day 180 of incubation) while LF only loses 5% of its initial mass during the whole period of incubation. Furthermore, even when UT reaches a high degree of disintegration, it has a much lower and slower degradation rate than GR.

**Figure 8:**
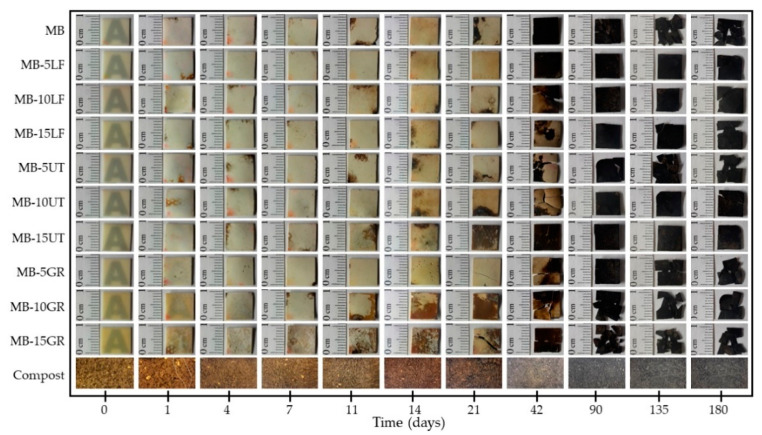
The visual aspect of MB and MB-pine resin derivatives film recovered at different incubation days under composting conditions and the evolution of compost medium during the test.

**Figure 9:**
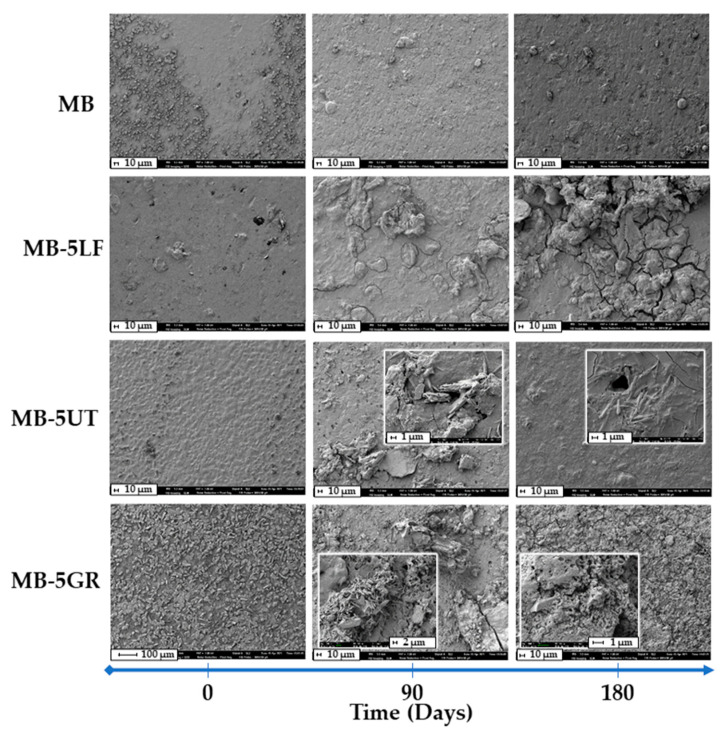
Scanning electron microscopy (SEM) images of the surface of films before (0) and after 90 and 180 days under composting conditions of MB and MB-pine resin derivatives.

Once the disintegration behavior of neat resins was studied, the compostability test of the MB-resin films was also performed. Figure 8 shows comparative pictures to qualitative see the visual appearance of the disintegrated film formulations, MB film, and MB- pine resin derivatives films, on different days of incubation under simulated composting conditions. It is observed that the opacity of the samples increases during the test, presenting noticeable changes from day 1 of incubation, where the samples lose transparency. On day 14, a yellowish coloration becomes noticeable for all samples and on day 90 the coloration of all formulations turns black. These changes in color and opacity indicate a change in the refractive index of the materials that may be due to hydrolytic degradation, the presence of water, and/or the presence of low molecular weight compounds formed by an enzymatic attack on the glycosidic bonds of the starch component of the Mater-Bi matrix [21,39,53]. Moreover, the conditions of the compost in which the samples were buried could also contribute to these color changes (see the compost visual appearance during the days of incubation). However, the loss of transparency is an indication that degradation under composting conditions begins in the amorphous part of the studied materials [39]. In Figure 8, it is seen that the fragmentation of the material is dependent on the type of additive in the blends. It is noted that for the blends containing GR, with the highest acid number, the fragmentation begins on day 21 of the test, while for those containing UT and LF, it begins on day 42. Neat MB matrix fragments between days 42 and 90. In fact, from SEM analysis (shown in Figure 9), it could be seen that at 90 days of disintegration under composting conditions, all MB-resin materials show advanced sign of microstructural disintegration, being particularly marked in MB blended with GR, in accordance with the degradation time of the neat GR resin (Figure 6). Meanwhile, neat MB microstructure show less degradation. These results show that pine resin derivatives delay the degradation and disintegration of the MB matrix under composting conditions because of the chemical interactions between UT or LF and MB polymeric matrix [30]. GR, on the contrary, establish fewer chemical interactions with the polymeric matrix and interact better with water due to the high availability of the hydroxyl groups and thus allowing greater mobility of the chains of the material [30], which facilitates the water penetration as well as the subsequent enzymatic degradation, showing advance microstructural disintegration at 180 days (as shown in Figure 9).

**Figure 10:**
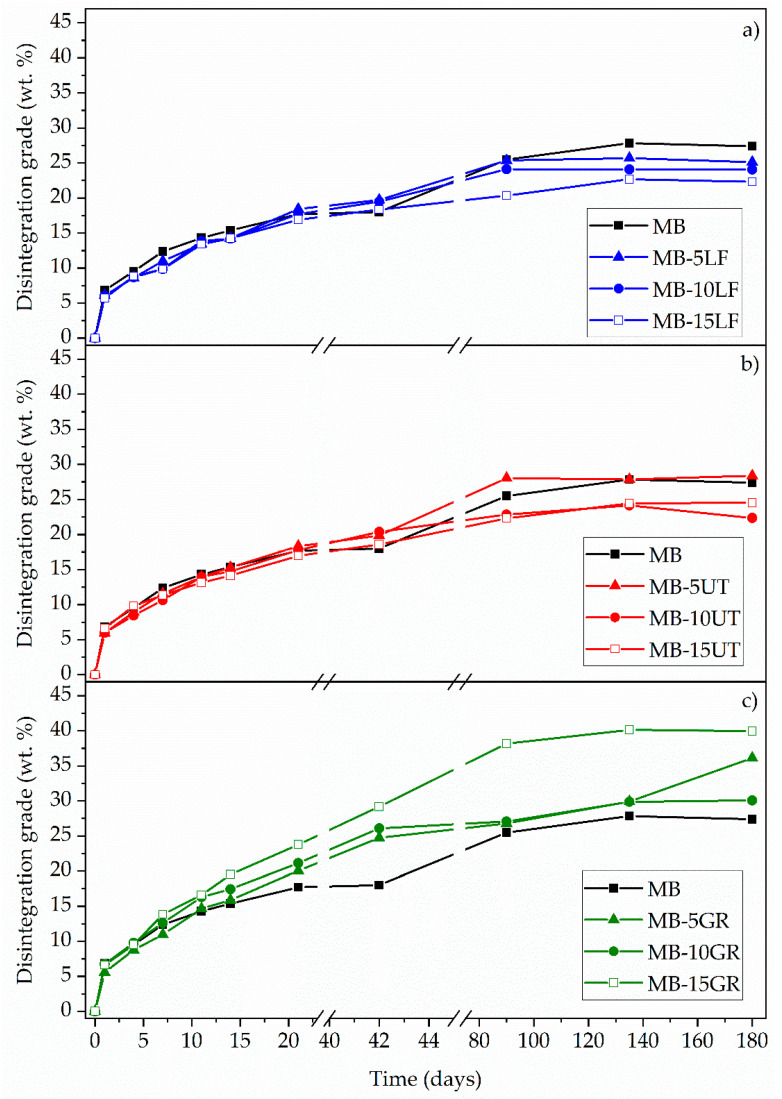
Disintegration degree of MB and MB-pine resin derivatives films at different days of extraction, under composting conditions for (**a**) MB-LF, (**b**) MB-UT and (**c**) MB-GR blends.

Figure 10 presents a quantitative measurement of the disintegration under composting conditions reported by the disintegration degree as a function of time for the MB and the formulations with pine resin derivatives. It can be noted that all the studied films have similar degradation kinetics, with slight variations towards the end of the test (180 days). The degree of disintegration has a progressive increase with the incubation time. Until day 21, the disintegration rate is high, reaching disintegration values between 15 and 20% for all materials. From day 21, the curve becomes asymptotic, showing a decrease in the degradation rate in all the studied formulations. Mater-Bi does not completely disintegrate during the 180 days of testing, reaching a maximum of 28% of disintegration. This result is closely related to that reported by Mohee et al. (2008), who obtained a 26.9% disintegration at 72 days, for a Mater-Bi type material [54].

According to Borchani et al. (2015), Mater-Bi (NF803, grade N) is constituted of 70 wt.% of PBAT, 20 wt.% of TPS, and 10 wt.% of PCL, and minor amounts of other additives [18]. Therefore, the disintegration time of Mater-Bi is affected by the disintegration time of the pure constitutive polymers. Sessini et al. (2019) found that a pure TPS matrix completely degrades in 60 days [55]. Other authors show that the maximum disintegration of TPS is between 73% [56] and 87% after 90 days of the test [57,58]. Literature also reports that PCL is completely degraded between 45 and 90 days [59,60]. However, PBAT disintegration is much slower and can take up to 180 days to disintegrate 90% [61]. Although other authors report that PBAT takes 230 days to reach just 35% disintegration, under the same composting conditions of this study (58 °C) since its structure must be hydrolyzed before microorganisms consume it as a source of nutrients [62]. Hence, the high content of PBAT (70%) in the matrix explains the low disintegration degree of the material.

The behavior of the formulations containing pine resin derivatives slightly varies concerning that of the MB matrix. In general, from day 90, the disintegration value remains constant for all formulations and increases towards day 180 for formulations containing GR (especially in MB-10GR and in MB-15GR) (Figure 10c). However, LF and UT decrease the degree of disintegration in the formulations in which they are present. Thus, pine resin derivatives influence the disintegration degree of Mater-Bi in two ways. On the one hand, the unmodified resin (GR) increases the degree of disintegration as its composition increases in the matrix. MB-15GR is the one with the highest degree of disintegration, 15% higher compared to the neat MB matrix, reaching a final degree of disintegration of 40% in 180 days of testing. On the other hand, the addition of UT or LF reduces the disintegration capacity of the material (reaching values between 20 and 25% of disintegration in 180 days of testing), due to the lower availability of hydrophilic groups given the modification of its structure [32], as discussed for the neat resins disintegration behavior. The disintegration differences between gum rosin and the pentaerythritol esters of gum rosin represent an advantage since they allow to have wider possibilities against the disintegration performance of the material, depending on the final material characteristics desired.

## 4. Conclusions

Mater-Bi^®^-based films were prepared by blending MB with pine resin derivatives, two pentaerythritol rosin esters (LF and UT) with different softening points and number acid, and neat gum rosin (GR). It was determined that pine resin derivatives were homogeneously distributed in MB films which provide homogeneity to the polymeric blend formulation, translucency, and uniform color. However, GR presents high color differences (yellowish), which can be a non-desired characteristic in food packaging applications. LF and UT in 5 and 10 wt.% present a total color difference smaller than 5 respect neat MB, which indicates that the color differences between these films cannot be detected by an inexperienced eye. But at higher contents of 15 wt.%, the color changes are too high. It was also found that all the pine resin additives provide a complete UV blocking effect in the UVB region, and a protective effect in the UVA region, which is directly dependent on the pine resin derivative content. Regarding the wettability of the films, it was determined that the addition of pine resin derivatives tends to increase the MB hydrophobicity, particularly UT and LF in 5 and 10 wt.% content. The oxygen transmission rate (OTR) analysis showed that oxygen permeability of the MB matrix was reduced when GR was incorporated, it was mainly maintained by UT in all the proportions and by LF at lower contents of additive. In contrast, higher LF contents (15 wt.%) produces a detriment to the MB oxygen barrier performance. It was observed that UT resin, with a lower softening point than LF, allowed obtaininment of a more homogeneous material since it was able to establish better interaction with MB polymeric matrix.

In the disintegration under composting condition analysis, it was concluded that the capacity of pine resin derivatives to be disintegrated under composting condition depends on its availability of the resin hydroxyl groups, which make them prone to hydrolytic degradation. Therefore, GR could be completely degraded in 90 days, while UT and LF degrade 90% and 5% respectively in 180 days of incubation. It was determined that MB and MB-pine resin derivatives present similar degradation rates. Besides, MB and MB- pine resin derivatives attain low levels of disintegration under composting conditions (30% to 40%), even after 180 days of incubation. The low disintegration degree was explained by the degradation time of the polymer components of MB, mainly due to PBAT which composition in the MB matrix is the highest (70%), and which disintegration time was reported to be higher than 230 days. Furthermore, it was noticed that the fragmentation time in MB can be tuned depending on the pine resin derivative used. On one hand, GR speeds up the hydrolytic MB degradation which further facilitates the enzymatic degradation and therefore the fragmentation takes place on day 21. On the other hand, UT and LF interact with MB hydrolytic groups delaying its fragmentation, which occurs between day 42 to 90.

These results obtained here suggest that Mater-Bi^®^ NF866 blended with pine resin derivatives are promising materials for films intended for food-related applications (food packaging and/or agricultural mulch films).

## Figures and Tables

**Table 1:** Coding of the studied materials according to the content and type of pine resin derivative.

Coding	Mater-Bi^®^ NF 866 Content (wt.%)	Resin Content (wt.%)	Type of Resin Added
MB	100	0	-
MB-5LF	95	5	Lurefor 125
MB-10LF	90	10	Lurefor 125
MB-15LF	85	15	Lurefor 125
MB-5UT	95	5	Unik Tack P100
MB-10UT	90	10	Unik Tack P100
MB-15UT	85	15	Unik Tack P100
MB-5GR	95	5	Gum rosin
MB-10GR	90	10	Gum rosin
MB-15GR	85	15	Gum rosin

**Table 2:** CIELa*b* coordinates values, yellow index, and total color differences values for the pine resin derivatives and the obtained films.

Coding	L	a*	b*	YI	∆E*_ab_^†^
Pine resin derivatives					
LF	48.35 ± 1.11 ^1^	1.49± 0.14 ^1^	18.05 ± 1.63 ^1^	41.15 ± 0.93 ^1^	40.79 ± 1.89
UT	47.92 ± 1.56 ^1^	−1.60 ± 0.27 ^2^	21.56 ± 0.68 ^2^	54.42 ± 1.74 ^2^	41.48 ± 1.61
GR	36.82 ± 0.79 ^2^	10.07 ± 1.51 ^3^	25.14 ± 1.87 ^3^	71.06 ± 2.58 ^3^	53.86 ± 2.35
Obtained films					
MB	89.04 ± 0.09 ^a^	0.25 ± 0.19 ^a^	16.34 ± 0.05 ^a^	30.69 ± 0.08 ^a^	-
MB-5LF	88.47 ± 0.27 ^a,b^	0.03 ± 0.11 ^a^	17.85 ± 0.17 ^b^	33.20 ± 0.45 ^b^	1.63 ± 0.23
MB-10LF	87.93 ± 0.56 ^b^	0.25 ± 0.48 ^a^	19.18 ± 0.42 ^c^	35.74 ± 1.22 ^c^	3.05 ± 0.67
MB-15LF	87.68 ± 0.19 ^b,c^	−0.19 ± 0.07 ^a^	21.15 ± 0.11 ^d^	38.66 ± 0.27 ^d^	5.02 ± 0.16
MB-5UT	88.23 ± 0.02 ^a,b^	−0.15 ± 0.09 ^a^	18.03 ± 0.11 ^b^	33.41 ± 0.24 ^b^	1.92 ± 0.13
MB-10UT	88.18 ± 0.02 ^b^	−0.21 ± 0.25 ^a^	20.71 ± 0.16 ^d^	37.76 ± 0.46 ^d^	4.48 ± 0.14
MB-15UT	87.06 ± 0.44 ^c,d^	−0.18 ± 0.29 ^a^	25.21 ± 0.29 ^e^	45.26 ± 0.84 ^e^	9.10 ± 0.44
MB-5GR	86.57 ± 0.22 ^d^	0.16 ± 0.09 ^a^	26.98 ± 0.15 ^f^	48.42 ± 0.24 ^f^	10.92 ± 0.18
MB-10GR	83.29 ± 0.08 ^e^	1.90 ± 0.17 ^a^	32.38 ± 0.59 ^g^	59.25 ± 0.78 ^g^	17.12 ± 0.54
MB-15GR	82.27 ± 0.41 ^f^	3.03 ± 0.02 ^a^	37.42 ± 0.41 ^h^	67.68 ± 0.38 ^h^	22.31 ± 0.51

^†^ ∆E* was calculated taking MB as reference. ^1–3^ Different numbers show statistically significant differences between the used additives (*p <* 0.05). ^a–h^ Different letters show statistically significant differences between the obtained films (*p <* 0.05).

## Data Availability

The data presented in this study are available on request from the corresponding author.

## Supplementary Materials

The following are available online at https://www.mdpi.com/article/10.3390/polym13091506/s1, Figure S1: Scanning electron microscopy (SEM) images of the cryofractures surface of Mater-Bi (MB) and MB with 5, 10 and 15 wt.% of pine resin derivatives (LF, UT and GR, respectively), Figure S2: Fourier transform infrared spectroscopy (FTIR) of Mater-Bi (MB) and MB with 15 wt.% of pine resin derivatives (LF, UT and GR, respectively) with expanded area between 1800 and 1650 cm-1, Figure S3: Scanning electron microscopy (SEM) images of the surface of films of Mater-Bi (MB) and MB with 5 wt.% of pine resin derivatives (LF, UT and GR).
