# Supplementary material for: Films Based on Mater-Bi® Compatibilized with Pine Resin Derivatives: Optical, Barrier, and Disintegration Properties
Source: Polymers (Basel). 2021 May 7;13(9):1506. doi: 10.3390/polym13091506 (PMC8124954; doi:10.3390/polym13091506)
Supplement: Supplementary file 1 [file polymers-13-01506-s001.zip › polymers-1166001-supplementary.pdf]

Supporting Information

Films based on Mater-Bi® compatibilized with pine resin derivatives: optical, barrier, and disintegration properties

Miguel Aldas[1,2*], Cristina Pavon[1], José Miguel Ferri[1], Marina Patricia Arrieta[3,4], Juan López-Martínez[1*]

[1] Instituto de Tecnología de Materiales (ITM), Universitat Politècnica de València (UPV), 03801 Alcoy, Spain; crisppavonv@gmail.com (CP), joferaz@upvnet.upv.es (JMF) jlopezm@mcm.upv.es (JL-M)

[2] Departamento de Ciencia de Alimentos y Biotecnología, Facultad de Ingeniería Química y Agroindustria, Escuela Politécnica Nacional, 170517 Quito, Ecuador; miguel.aldas@epn.edu.ec (MA)

[3] Departamento de Ingeniería Química y del Medio Ambiente, Escuela Técnica Superior de Ingenieros Industriales, Universidad Politécnica de Madrid (ETSII-UPM), Calle José Gutiérrez Abascal 2, 28006, Spain; m.arrieta@upm.es (MPA)

[4] Grupo de Investigación: Polímeros, Caracterización y Aplicaciones (POLCA), 28006 Madrid, Spain

* Correspondence: (MA) miguel.aldas@epn.edu.ec, Tel.: +593 999 736 444 ; (JL-M) jlopezm@mcm.upv.es, Tel.: +34 654 172 022;

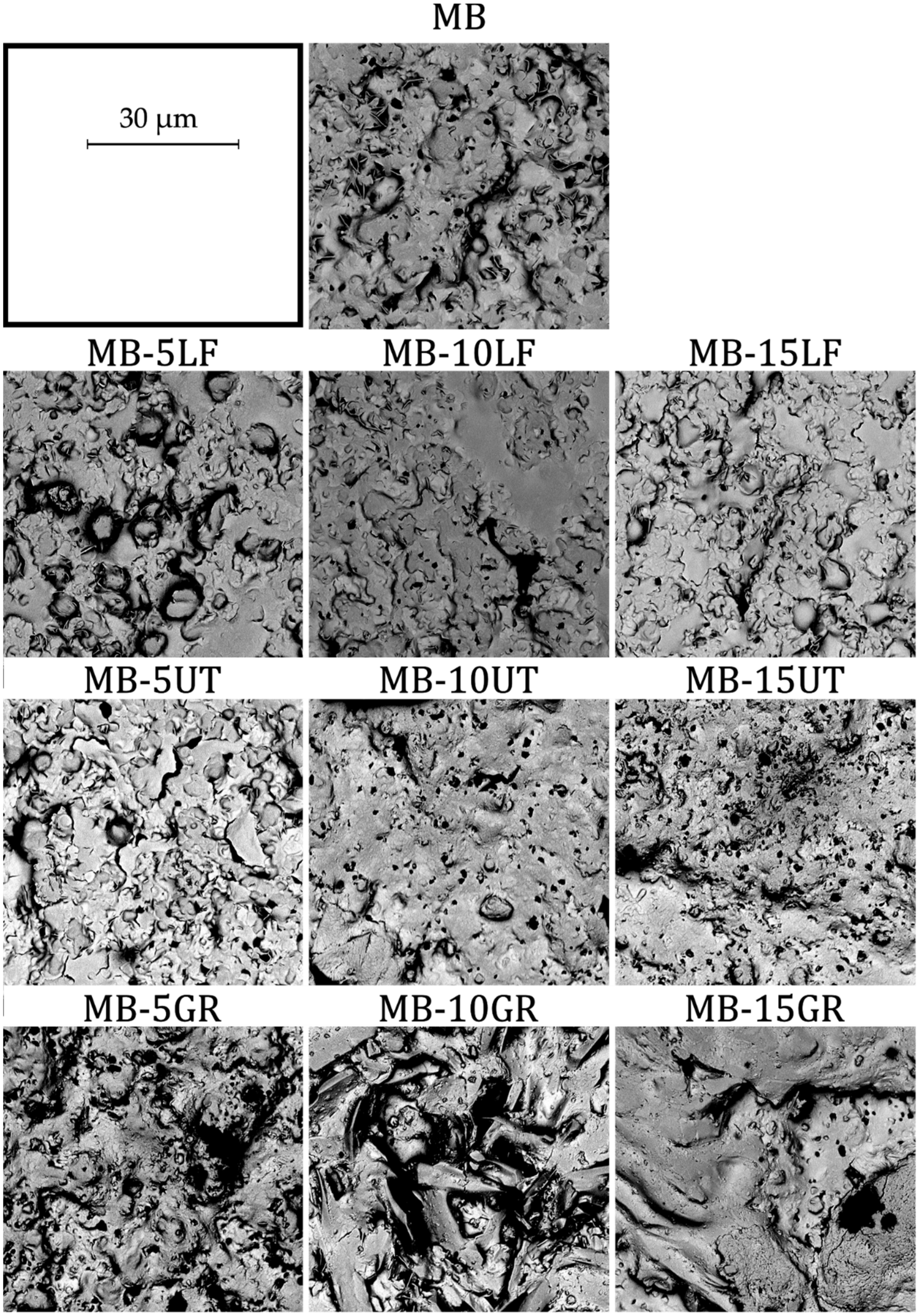

Figure S1. Scanning electron microscopy (SEM) images of the cryofractures surface of Mater-Bi (MB) and MB with 5, 10 and 15 wt.% of pine resin derivatives (LF, UT and GR, respectively)

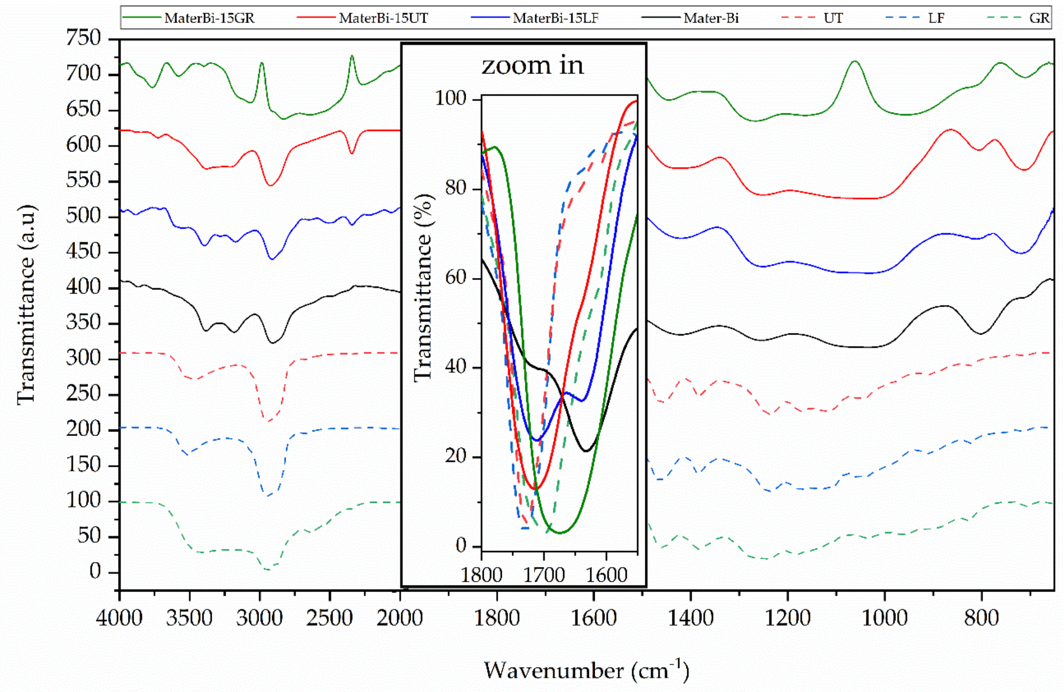

Figure S2. Fourier transform infrared spectroscopy (FTIR) of Mater-Bi (MB) and MB with 15 wt.% of pine resin derivatives (LF, UT and GR, respectively) with expanded area between 1800 and 1650 cm^{-1}

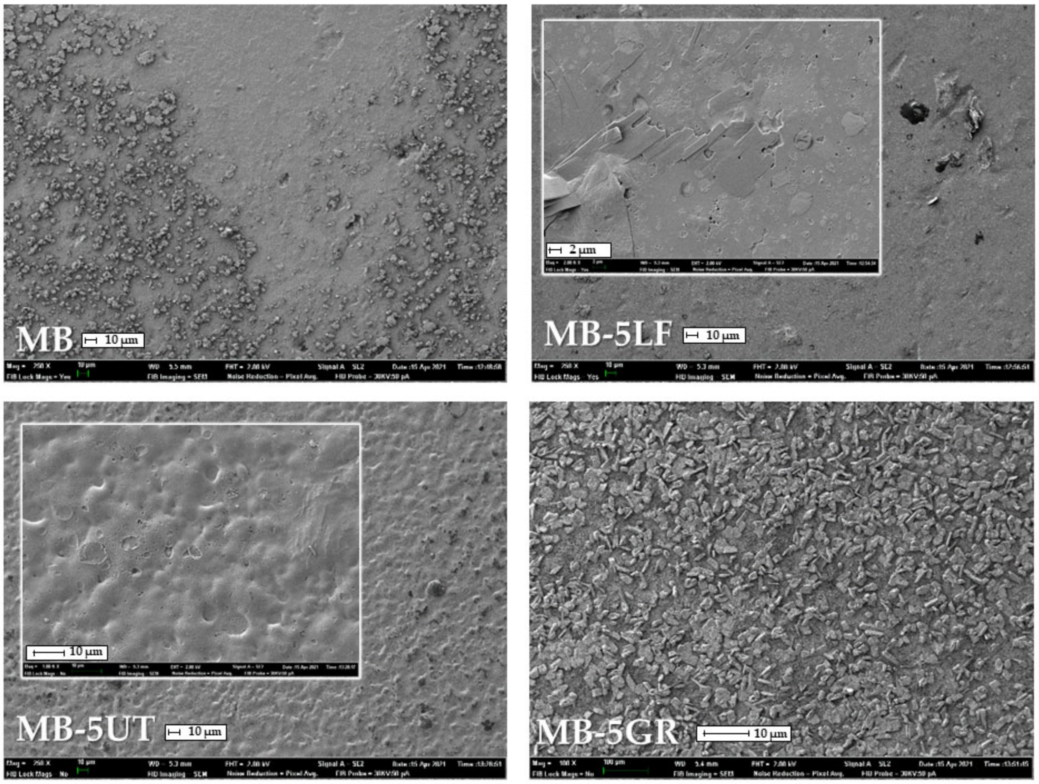

Figure S3. Scanning electron microscopy (SEM) images of the surface of films of Mater-Bi (MB) and MB with 5 wt.% of pine resin derivatives (LF, UT and GR)
